# Marginal gap formation and fracture strength of indirect inlay restorations cemented with preheated resin composites: an in vitro study

**DOI:** 10.1186/s12903-026-09466-1

**Published:** 2026-08-01

**Authors:** Engin Işık, Evrim Eliguzeloglu Dalkilic

**Affiliations:** 1 Department of Restorative Dentistry, School of Dental Medicine, Bahçeşehir University, Istanbul, Turkey; 2 Department of Restorative Dentistry, Faculty of Dentistry, Bezmialem Vakıf University, Istanbul, Turkey

**Keywords:** CAD/CAM, Fracture strength, Gap formation, Indirect restoration, Inlay, Preheated resin composite

## Abstract

**Background:**

Optimal marginal adaptation and adequate fracture strength are critical for the long-term clinical success of indirect posterior restorations. Preheated resin composites have been proposed as alternative luting agents; however, their effects on marginal integrity and mechanical performance of inlays remain unclear. This in vitro study aimed to evaluate gap formation and fracture strength of indirect inlay restorations cemented with preheated resin composites compared with a conventional resin cement.

**Methods:**

Forty human mandibular molar teeth were divided into four groups (*n* = 10/group). Group 1 used a conventional dual-cure resin cement. Group 2 used a preheated resin composite. Group 3 used a preheated bulk-fill resin composite. Group 4 served as the intact tooth control group and received no cavity preparation or restoration. Standardized Class II MOD inlay preparations were performed, with the mesial proximal box floor located in enamel and the distal box floor located in dentin. Indirect restorations were fabricated from resin nano-ceramic blocks via computer-aided design and computer-aided manufacturing (CAD-CAM). All teeth underwent thermal cycling and chewing simulation. Marginal gap formation was analyzed using micro-computed tomography (micro-CT). Fracture strength was assessed using a universal testing machine, and fracture modes were classified. Data were analyzed using the Shapiro–Wilk test and Levene’s test, followed by one-way ANOVA and repeated measures ANOVA with Bonferroni correction and Tukey’s post hoc test, and the Fisher–Freeman–Halton test.

**Results:**

Enamel margin gap volumes were significantly higher in the preheated bulk-fill composite group compared with the conventional resin cement group (*p* = 0.012), while no significant differences were observed at dentin margins (*p* = 0.182). All restored groups exhibited significantly lower fracture strength values compared to the intact teeth group (*p* = 0.003). Fracture mode distribution showed a tendency toward more repairable fractures in the preheated bulk-fill composite group and more irreparable fractures in the conventional resin cement group.

**Conclusions:**

Preheated resin composites showed comparable performance to conventional resin cement in terms of marginal adaptation and fracture strength. However, their performance appears to be material-dependent. Therefore, preheated resin composites may be considered as potential alternative luting materials for indirect restorations within the limitations of this study.

**Clinical relevance:**

Preheated resin-based composites are increasingly considered as alternative luting agents for indirect restorations. Understanding their influence on marginal adaptation and mechanical performance may assist clinicians in selecting appropriate cementation strategies for indirect inlay restorations.

## Introduction

Indirect posterior inlay restorations remain a widely used and clinically relevant treatment option in contemporary restorative dentistry, offering a conservative alternative to full-coverage crowns when sufficient tooth structure is preserved. Medium- to long-term clinical studies and systematic reviews have reported survival rates commonly ranging between approximately 85% and 97%, depending on restorative material, tooth vitality, and follow-up duration [1–5]. The increasing integration of digital workflows and CAD/CAM technologies has further expanded the clinical use of inlays by enabling predictable chairside fabrication using contemporary glass-ceramic, polymer-infiltrated ceramic, and resin-matrix ceramic materials [1, 2, 6]. Despite favorable overall outcomes, clinical performance remains influenced by factors such as remaining tooth structure, cuspal coverage requirements, and vitality status, underscoring the importance of optimized adhesive and luting strategies for long-term success [4, 5]. 

Polymerization shrinkage represents one of the primary causes of marginal gap formation between the restorative material and the tooth, and the choice of luting agent is critical in modulating interfacial gap volumes [7–10]. Resin-based composites have been investigated as alternative luting agents for indirect restorations, particularly when used in conjunction with preheating [11–13]. Among these materials, high-viscosity bulk-fill resin composites have attracted increasing interest as luting agents for indirect restorations. Experimental studies have demonstrated favorable bond strength and mechanical performance when these materials are preheated and used beneath CAD/CAM restorations, supporting their potential as alternatives to conventional resin cements [14]. Preheating of resin composites has been proposed as a technique to enhance handling characteristics and potentially improve clinical outcomes by reducing viscosity, increasing flowability, and modifying polymerization behavior [12, 13, 15, 16]. The reduction in viscosity associated with preheating has been documented across different investigations and is attributed to increased polymer chain mobility at elevated temperatures, which facilitates resin flow and may improve adaptation to cavity walls and restoration internal surfaces [12, 17]. Recent physicochemical evaluations have shown that preheated resin composites maintain favorable mechanical stability and can contribute to strengthening of luted feldspathic ceramics [12, 16]. However, the effect of preheating on seating accuracy remains equivocal; one in vitro study reported that preheating did not reliably prevent incomplete seating of CAD/CAM inlays and onlays, suggesting that viscosity reduction alone may not guarantee improved marginal adaptation under clinical constraints [15]. These findings highlight the need for careful consideration of material properties, restoration geometry, and polymerization protocols when applying preheating techniques to luting procedures.

While the luting material directly affects gap formation at the tooth–restoration interface, it also plays a decisive role in stress distribution and the mechanical support of indirect restorations. Accordingly, fracture strength has become one of the principal outcomes used to evaluate the mechanical performance of indirect CAD/CAM inlay restorations, as it reflects the combined influence of restorative material, preparation design, and luting strategy under simulated functional loading [18, 19]. Highly filled resin composites offer several theoretical advantages over conventional dual-cure resin cements, including greater inorganic filler loading, which is associated with increased stiffness and enhanced mechanical support of the overlying restoration [13, 16]. The increased filler content of resin composites is hypothesized to improve rigidity and load transmission, potentially reducing stress concentration at the restoration-cement interface [13, 16]. However, comparative studies have yielded mixed results, with some investigations reporting superior fracture strength for dedicated resin cements over preheated resin composites, depending on restoration type, ceramic material, and cementation protocol [12, 20]. 

The aim of this in vitro study was to evaluate the marginal gap formation at enamel and dentin margins and the fracture strength of inlay restorations luted with preheated resin composite compared with conventional dual-cure resin cement. The following null hypotheses were tested: (1) there is no significant difference in marginal gap width at enamel and dentin margins between inlays luted with preheated resin composite and those luted with conventional dual-cure resin cement; (2) there is no significant difference in fracture strength between inlays luted with preheated resin composite and those luted with conventional dual-cure resin cement.

## Methods

### Specimen number and tooth selection

An a priori power analysis was performed using G*Power software (version 3.1.9.7; Heinrich Heine University Düsseldorf, Düsseldorf, Germany) based on a one-way analysis of variance (ANOVA) with four experimental groups [21]. Assuming an effect size (f) of 0.40, a significance level (α) of 0.05, and a desired statistical power (1 − β) of 0.80, the analysis indicated that a minimum of seven specimens per group was required. To compensate for potential specimen loss during specimen preparation and to increase the robustness of the statistical comparisons, 10 specimens per group were included, resulting in a total of 40 specimens.

40 human mandibular molars extracted for orthodontic or periodontal reasons were collected from patients treated at the Department of Oral and Maxillofacial Surgery, Faculty of Dentistry, Bezmialem Vakıf University. Each tooth was examined under x2.5 magnification using dental loupes and the dental operating light. A sharp dental explorer was used to confirm the absence of caries, restorations, visible cracks, and hypoplastic defects. Mesiodistal and buccolingual crown dimensions at the cementoenamel junction (CEJ) were measured using a digital caliper (accuracy: 0.01 mm), and only teeth with similar crown morphology, measuring approximately 10.5 mm mesiodistally and 9.0 mm buccolingually (maximum deviation: 10%), were included to minimize anatomical variability among specimens. The materials used in this study, together with their manufacturers and key components, are listed in Table 1.

**Table 1:** Materials used in the study

Material	Lot No.	Type	Composition
Grandio Blocs VOCO GmbH, Cuxhaven, Germany	2,137,181	Resin nano-ceramic CAD/CAM block	UDMA/DMA-based resin matrix reinforced with nano-hybrid silica and barium-aluminum-borosilicate glass fillers (86 wt% inorganic filler).
Futurabond U VOCO GmbH	2,109,068	Universal adhesive	Functional adhesive monomers (10-MDP), methacrylate monomers (HEMA, Bis-GMA), acidic functional monomers, silica nanofillers, ethanol, water, and photoinitiators.
Ceramic Bond VOCO GmbH	2,141,131	Silane coupling agent	Pre-hydrolyzed silane coupling agent dissolved in an organic solvent system.
Vococid VOCO GmbH	2,105,038	Phosphoric acid gel	37% phosphoric acid, silica thickening agents, and water.
Bifix Hybrid Abutment VOCO GmbH	2,124,630	Dual-cure resin cement	Multifunctional methacrylate monomers, inorganic fillers, dual-cure initiator system, stabilizers, and pigments.
VisCalor Bulk VOCO GmbH	2,118,759	Thermo-viscous bulk-fill resin composite	Bis-GMA-free UDMA-based resin matrix, inorganic glass/silica fillers, and photoinitiator system.
Filtek™ One Bulk-Fill Solventum, St. Paul, MN, USA	NC92633	Bulk-fill resin composite	AUDMA/AFM-based resin matrix, zirconia/silica nanocluster fillers, and ytterbium trifluoride radiopaque fillers.

*Abbreviations: *UDMA* urethane dimethacrylate, *DMA *dimethacrylate, *10-MDP* 10-methacryloyloxydecyl dihydrogen phosphate, *HEMA* 2-hydroxyethyl methacrylate, *Bis-GMA *bisphenol A-glycidyl dimethacrylate, *AUDMA* aromatic urethane dimethacrylate, *AFM* addition-fragmentation monomer

## Specimen preparation and grouping

Following extraction, teeth were cleaned with a hand scaler to remove residual soft tissue and calculus, polished with pumice, disinfected with chloramine-T trihydrate solution according to ISO specifications, and stored in distilled water until use. All teeth were embedded in cold acrylic resin (Paladent RR; Kulzer GmbH, Hanau, Germany) cylinders (3 cm diameter, 2 cm depth) using silicone molds, ensuring that the resin surface was 2 mm apical to the cementoenamel junction (CEJ) and that the long axis of the tooth was perpendicular to the base. Specimens were stored in distilled water until preparation.

Following specimen embedding, the teeth were numbered sequentially and randomly assigned to four groups (*n* = 10 each) using a computer-generated randomization sequence generated with the RAND function in Microsoft Excel (Microsoft Corp., Redmond, WA, USA):

- Group 1 (Conventional Resin Cement): Inlays cemented with a conventional dual-cure resin cement (Bifix Hybrid Abutment; VOCO GmbH, Cuxhaven, Germany).
- Group 2 (Preheated Resin Composite): Inlays cemented with a preheated resin composite (VisCalor Bulk; VOCO GmbH, Cuxhaven, Germany), preheated using a composite dispenser (VisCalor Dispenser; VOCO GmbH, Cuxhaven, Germany).
- Group 3 (Preheated Bulk-Fill Resin Composite): Inlays cemented with a preheated bulk-fill resin composite (Filtek One Bulk Fill; Solventum, St. Paul, MN, USA), preheated using the same composite dispenser.
- Group 4 (Control): Intact teeth with no cavity preparation or restoration.

The selection of preheated composite materials was influenced by the technical compatibility requirements of the heating device, which is designed to accommodate compule-based composite systems. Therefore, materials with compatible delivery formats were selected, and heating protocols were applied according to the respective manufacturer recommendations, resulting in different heating modes for each material.

### Cavity preparations

**Fig. 1:**
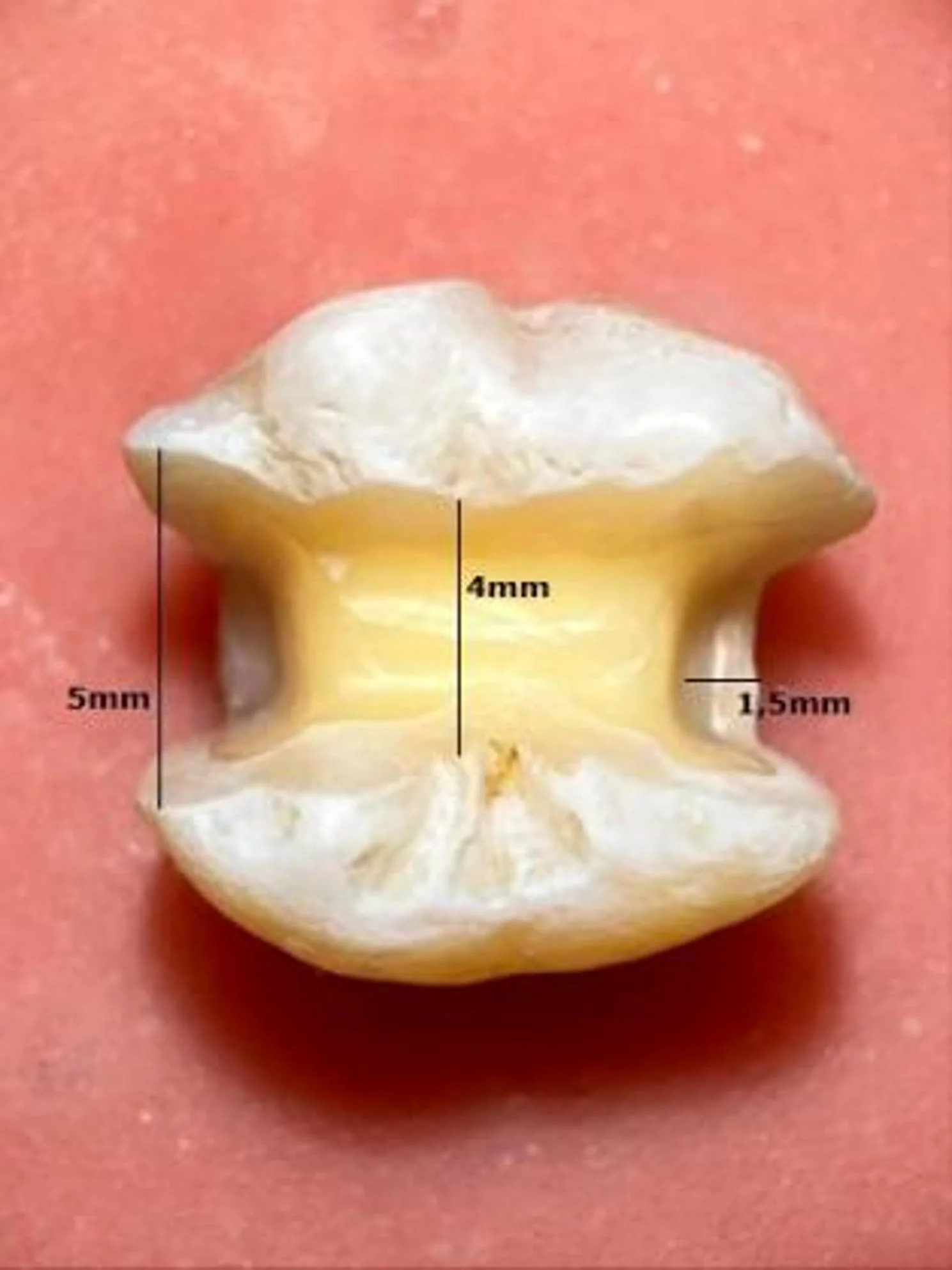
Occlusal view of cavity preparation

Standardized Class II MOD inlay cavities were prepared using cylindrical and tapered diamond burs (Meisinger GmbH, Neuss, Germany) under water cooling to achieve slightly divergent walls and a butt-joint design. Cavity dimensions were standardized according to predetermined measurements and the taper was achieved by the geometry of the tapered diamond burs, while cavity dimensions were periodically verified using a periodontal probe. The occlusal isthmus width was 4.0 mm, depth 3.0–3.5 mm, and proximal box width 5.0 mm, with an axial pulpal depth of 1.5 mm. Mesial proximal box floor was prepared in enamel (1.0–1.5 mm above the CEJ), and the distal box floor prepared in dentin (1.0–1.5 mm below the CEJ). Internal line and point angles were rounded (Fig. 1).

### Digital impression and indirect restoration fabrication

Digital impressions were taken using a CAD/CAM system (Cerec MC, Sirona Dental Systems, Bensheim, Germany) and Cerec Sw 5.2.3 software. The inlays were digitally designed using the CAD module of the software prior to milling and the cement space parameter was set to 100 μm. All inlays were milled from resin nano-ceramic blocks (Grandio Blocs; VOCO GmbH, Cuxhaven, Germany) using the Cerec milling unit. After milling, all restoration bonding surfaces were sandblasted with aluminum oxide (25–50 μm) at 1.5-2.0 bar according to the manufacturer’s instructions. Fabricated inlays were stored in numbered compartments until cementation. Inlay fit was assessed visually from mesial and distal aspects before cementation.

### Cavity restoration procedures

For tooth surface preparation, cavities were rinsed and dried. 37% phosphoric acid gel (Vococid, VOCO GmbH, Cuxhaven, Germany) was applied to enamel margins for 30 s and dentin surfaces for 15 s, followed by rinsing with water spray, blotting dry with cotton pellets, and air-drying for 5 s. A dual-cure universal bond (Futurabond U, VOCO GmbH, Cuxhaven, Germany) was then actively applied to all cavity surfaces for 20 s, air-thinned for 5 s, and light-cured for 20 s using a cordless light-curing unit (VALO; Ultradent Products Inc., South Jordan, UT, USA) in standard mode (approximately 1000 mW/cm²). Bonding surfaces of inlays were treated with silane (Ceramic Bond, VOCO GmbH, Cuxhaven, Germany) for 60 s, followed by 5 s of air-drying.

Following surface preparation, the respective luting material was applied to the cavity, and the inlay was seated under gentle finger pressure. After seating of the restoration, excess material was removed using a microbrush prior to light curing. Light curing was performed using the VALO cordless light-curing unit in standard mode from the occlusal, mesial, and distal aspects for 20 s each to ensure adequate light exposure at both occlusal and proximal regions.

A summary of the restorative and luting procedures for all experimental groups is presented in Table 2. Heating protocols differed between materials and were applied according to manufacturer recommendations, based on device compatibility requirements and material-specific guidelines, with Program 1 used for VisCalor Bulk and Program 2 for Filtek One Bulk Fill.

**Table 2:** Experimental groups and luting/restorative procedures

Group (*n* = 10)	Luting/Restorative Material	Application Protocol
Group 1 (Conventional Resin Cement)	Luting material: Bifix Hybrid Abutment (Dual-cure resin cement, VOCO GmbH, Cuxhaven, Germany)	Inlays were luted using a dual-cure resin cement without preheating, following standardized procedures.
Group 2 (Preheated Resin Composite)	Luting material: VisCalor^®^ Bulk resin composite (VOCO GmbH)	Inlays were luted using a resin composite, with the material preheated in a VisCalor^®^ Dispenser, Program 1 (30 s to 65 °C, maintained for 2.5 min), following standardized procedures.
Group 3 (Preheated Bulk-Fill Resin Composite)	Luting material: Filtek One Bulk-Fill resin composite (Solventum, St. Paul, MN, USA)	Inlays were luted using a bulk-fill resin composite, with the material preheated in VisCalor^®^ Dispenser, Program 2 (70 s to 65 °C, maintained for 1 min 50 s), following standardized procedures.

*Heating protocols differed between materials according to manufacturer recommendations and device compatibility requirements

### Thermal and mechanical aging

All restored specimens were subjected to thermal and mechanical aging:

1. Chewing Simulation: Specimens were placed in a chewing simulator (CS-4; SD Mechatronik GmbH, Feldkirchen-Westerham, Germany) and subjected to 50,000 chewing cycles at 100 N vertical force and 1.7 Hz frequency. Force was applied at the central fossae of restorations using 3.2 mm steel balls, and the simulation was performed in distilled water.
2. Thermal Cycling: Following chewing simulation, specimens underwent 10,000 thermal cycles in a thermocycler (THE-1100; SD Mechatronik GmbH, Feldkirchen-Westerham, Germany) between 5 °C and 55 °C, with a 30 s dwell time and 10 s transfer time.

## Micro-CT analysis (Gap formation)

For gap formation analysis, 15 randomly selected specimens (five from each of restored groups) were analyzed using a micro-computed tomography system (SkyScan 1172; Bruker microCT, Kontich, Belgium). Scans were performed at 88 kV/112 µA with 0.6° rotational steps, random movement of 20, and an average frame of 5, using an Al + Cu filter at 1 K (668 × 1000). The exposure time was 335 ms, and the pixel size was 17–18 μm. Data were reconstructed with NRecon software and analyzed using CTAn (Version 1.16.4.1; Skyscan, Kontich) to calculate gap volumes from standardized regions above the gingival margins at both mesial and distal surfaces. Gap analysis was based on the detection of black spaces within the volume of interest (VOI), derived from two-dimensional slices in the region of interest (ROI). VOIs were delineated around the restorative margins, and three-dimensional images were generated using CTvox (Version 3.1.1 r1191; Skyscan, Kontich). The gap volume at the tooth-restoration interface was calculated and reported in mm³.

### Fracture strength and fracture mode analysis

**Fig. 2:**
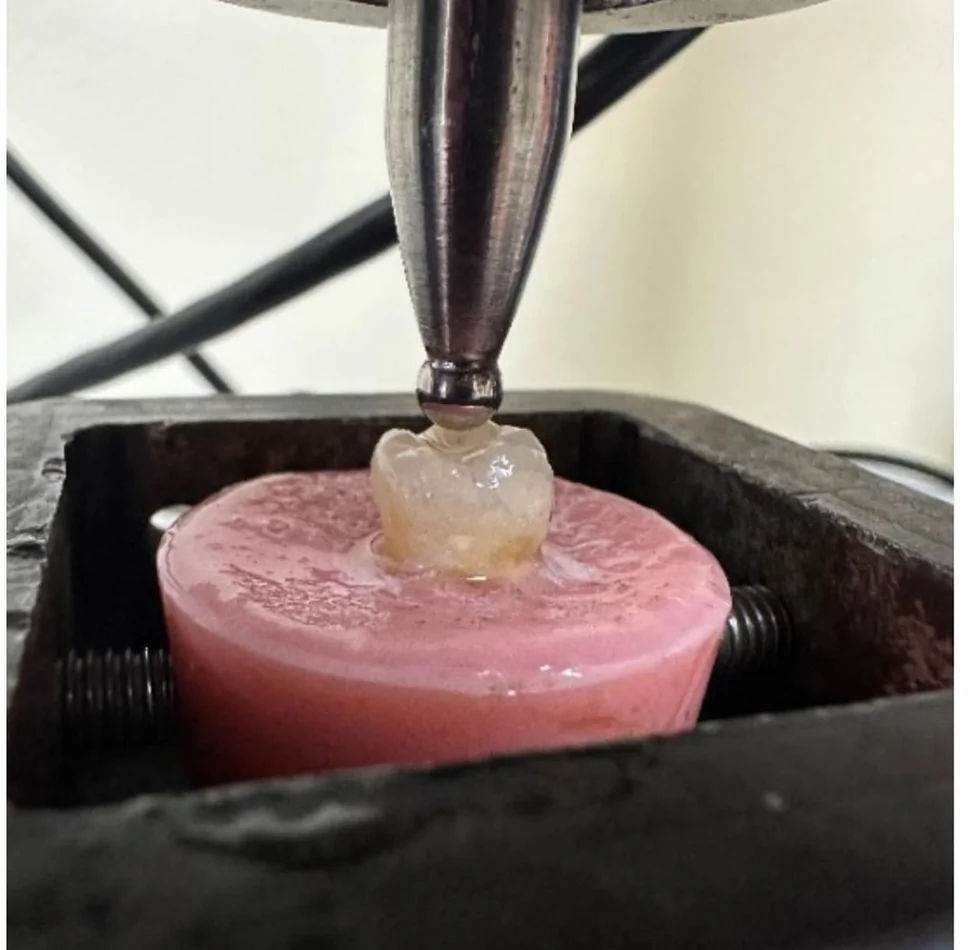
5 mm diameter steel ball of the universal test machine

All specimens were subjected to fracture strength testing using a universal testing machine (AGS-X; Shimadzu Corporation, Kyoto, Japan). A 5 mm diameter steel ball applied force to the central fossae of the teeth at a crosshead speed of 0.5 mm/s until fracture (Fig. 2). Fracture strength values were recorded in Newtons (N).

Fractured specimens from restored groups were examined and photographed using light microscopy (SMZ1000; Nikon Corporation, Tokyo, Japan) at 0.8x magnification. Modes of fracture were classified according to Burke [22]:

- Mode I (Repairable): Isolated restoration fracture confined within cavity margins.
- Mode II (Repairable): Restoration fracture involving a small tooth section.
- Mode III (Repairable): Restoration fracture accompanied by tooth fracture above the CEJ.
- Mode IV (Irreparable): Restoration fracture accompanied by tooth fracture below the CEJ.

### Statistical analysis

Data were analyzed using the Shapiro–Wilk test and Levene’s test to assess normality and homogeneity of variances, respectively. For the micro-CT gap formation analysis, intergroup comparisons were performed using one-way ANOVA, while mesial–distal margin comparisons were analyzed with repeated measures ANOVA followed by Bonferroni correction. For the fracture strength analysis, intergroup differences were evaluated using one-way ANOVA with Tukey’s post hoc test. For the fracture mode analysis, categorical data were analyzed using the Fisher–Freeman–Halton test.

## Results

### Gap formation (Micro-CT analysis)

**Fig. 3:**
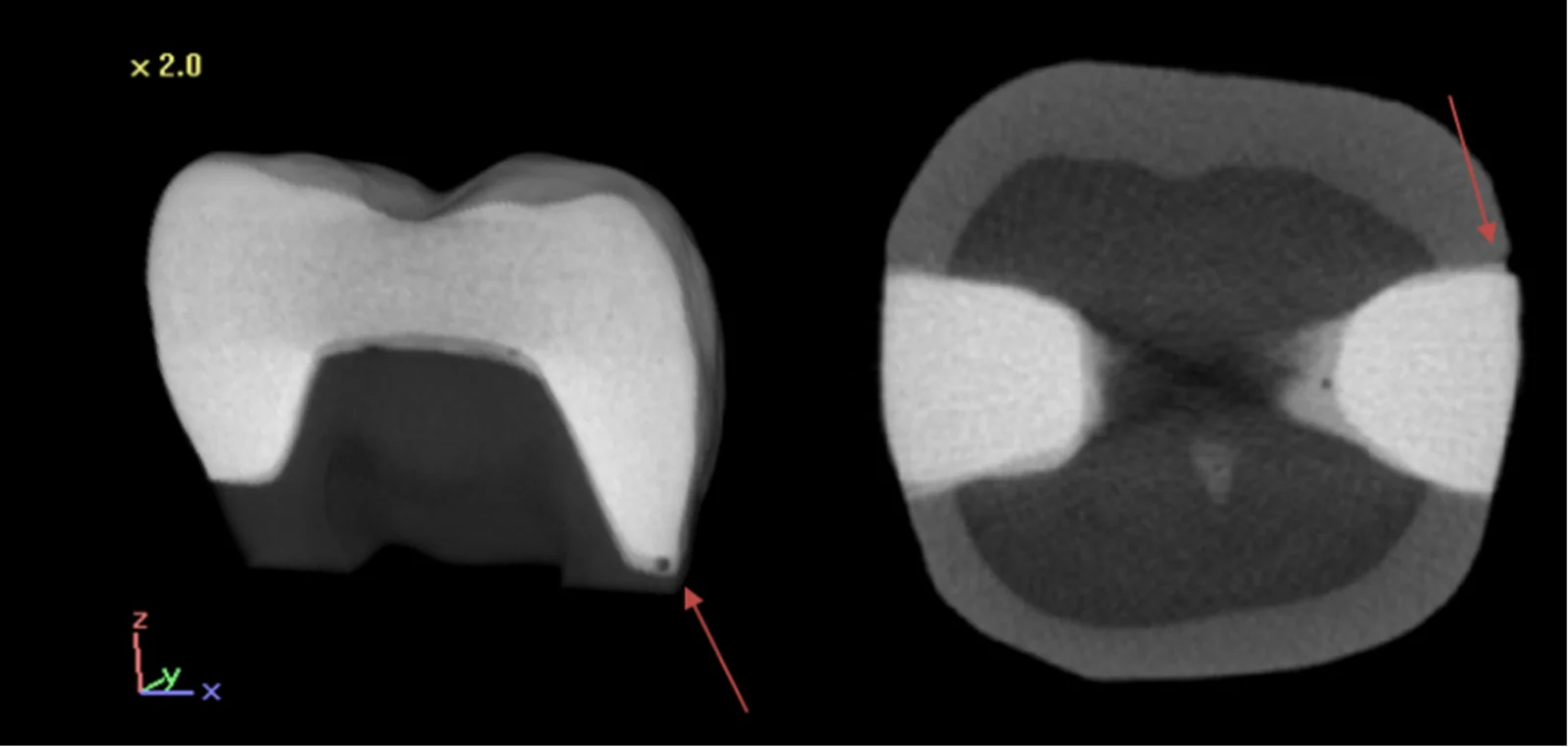
Representative micro-CT image of group 1 (arrows indicate gaps)

**Fig. 4:**
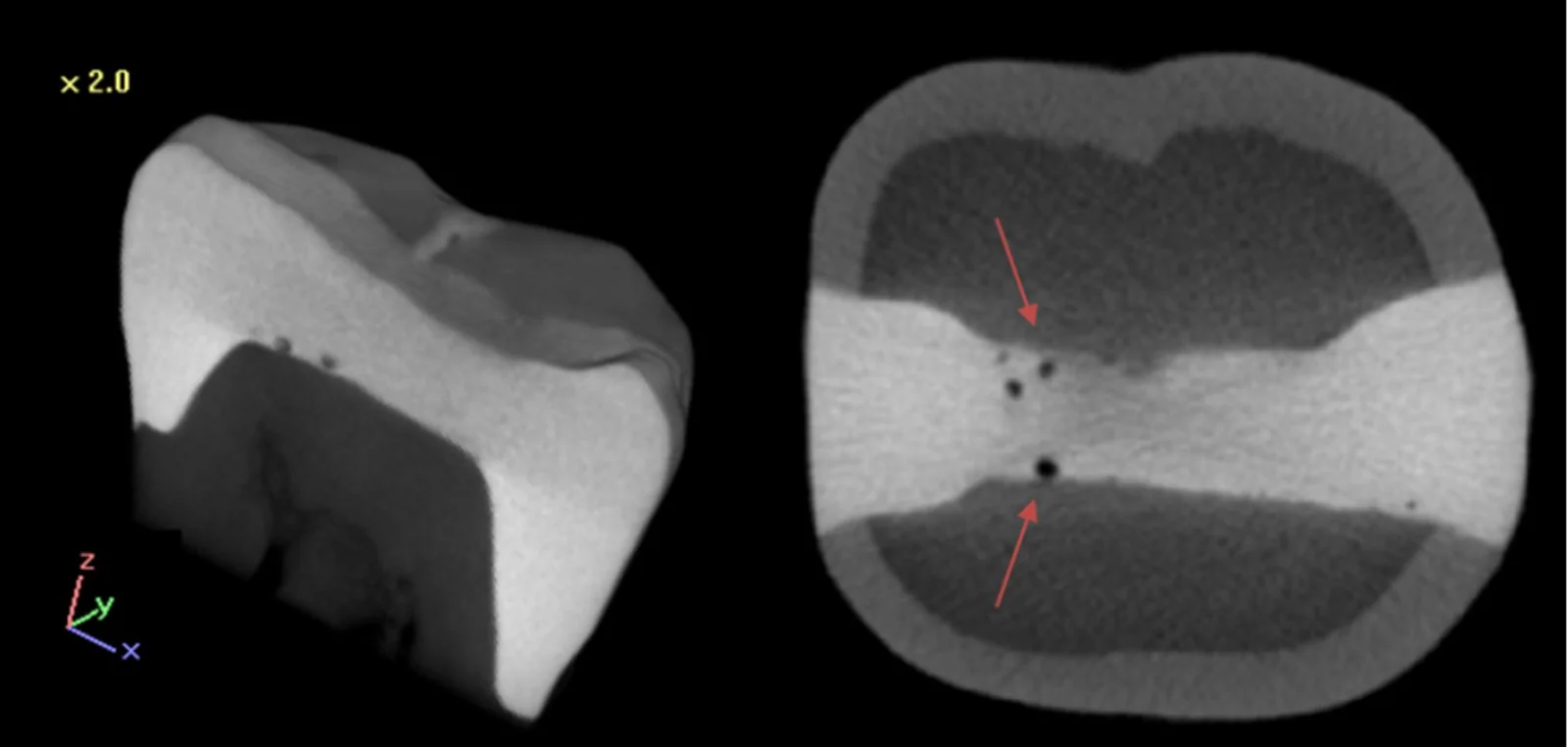
Representative micro-CT image of group 2 (arrows indicate gaps)

**Fig. 5:**
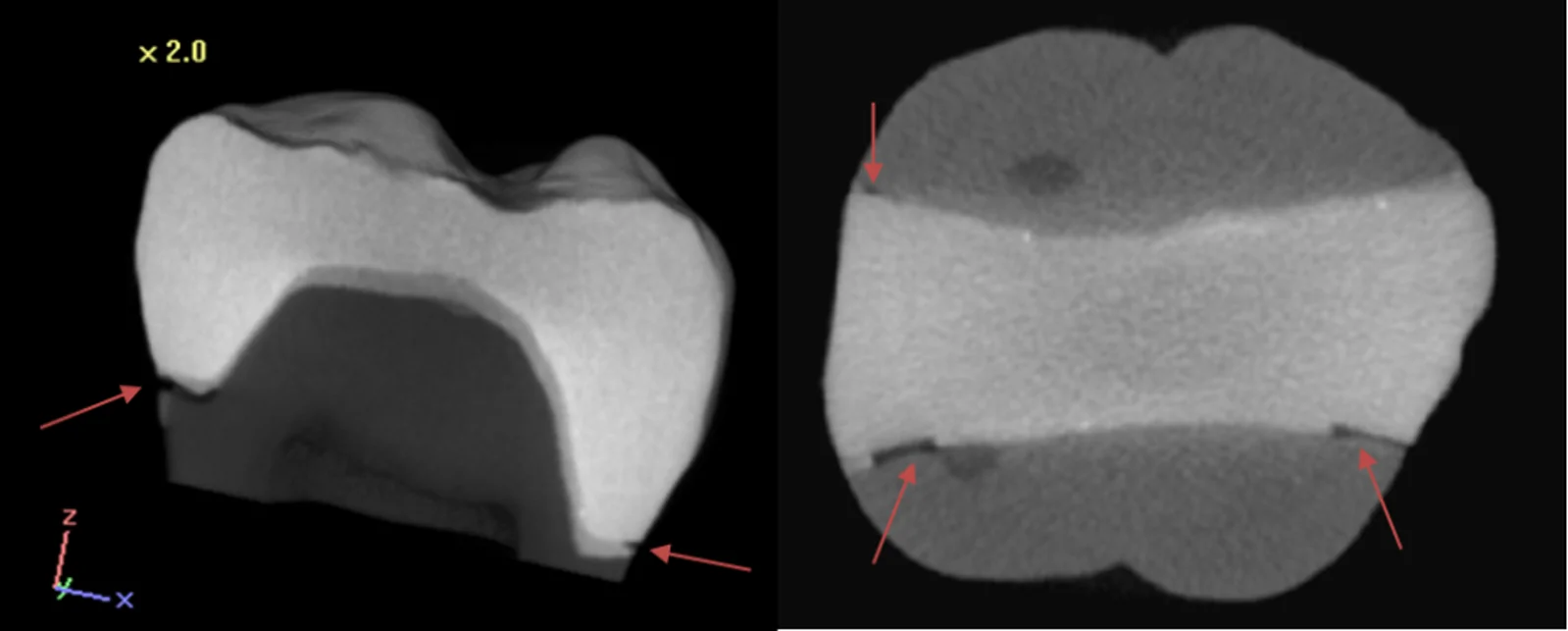
Representative micro-CT image of group 3 (arrows indicate gaps)

The gap formation results obtained by micro-CT are presented in Table 3, with representative images shown in Figs. 3, 4 and 5. The enamel margin values of Group 3 showed the highest gap volume, while those of Group 1 were the lowest; the difference between these two groups was statistically significant (*p* = 0.012). Groups 1 and 2 were statistically similar (*p* = 0.874). For dentin margins, all groups showed comparable values (*p* = 0.182). In Group 1, enamel margin values were significantly lower than dentin margin values (*p* < 0.001), whereas in groups 2 and 3, enamel and dentin margin values were statistically similar (*p* = 0.131 and *p* = 0.720, respectively).

**Table 3:** Mean gap formation values, standard deviations, and intergroup differences at the enamel and dentin margins

Group (*n* = 5)	Enamel margin (mm^3^)	Dentin margin (mm^3^)	*p* value
Group 1	0.162 (0.044) a	0.448 (0.203)	< 0.001
Group 2	0.223 (0.065) a	0.329 (0.058)	0.131
Group 3	0.572 (0.326) b	0.596 (0.301)	0.720
p value	0.012	0.182	

*Different letters indicate statistically significant differences among groups (*p* ≤ 0.05)

### Fracture strength

Fracture strength values are summarized in Table 4. Significant differences were found among the groups (ANOVA, *p* = 0.003). The intact control group (Group 4) showed significantly higher fracture strength than all restored groups (Groups 1–3).

**Table 4:** Mean fracture strength values, standard deviations, 95% confidence intervals and intergroup differences

Group (*n* = 10)	Mean fracture strength (*N*)	95% CI (*N*)
Indirect Inlay - Conventional Resin Cement	2021.81 (522.53) a	1648.01–2395.60
Indirect Inlay - Preheated Resin Composite	2093.08 (598.42) a	1664.99–2521.16
Indirect Inlay - Preheated Bulk-Fill Resin Composite	2141.68 (444.06) a	1824.01–2459.34
Intact Teeth (Control)	2905.15 (492.69) b	2552.70–3257.60

* Different letters indicate statistically significant differences among groups (*p* = 0.003)

### Fracture mode distribution

**Fig. 6:**
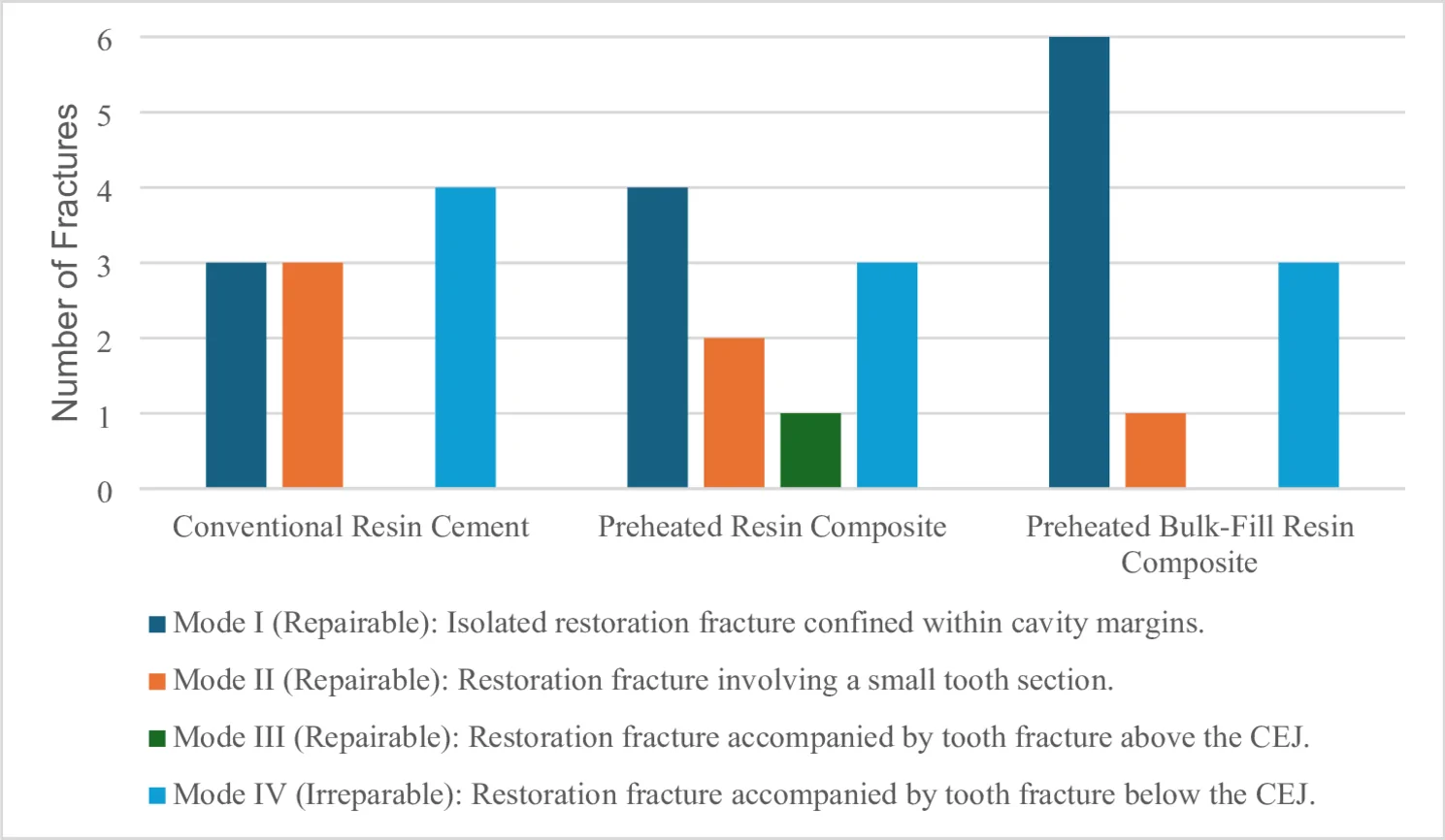
Distribution of fracture modes among the tested groups

**Fig. 7:**
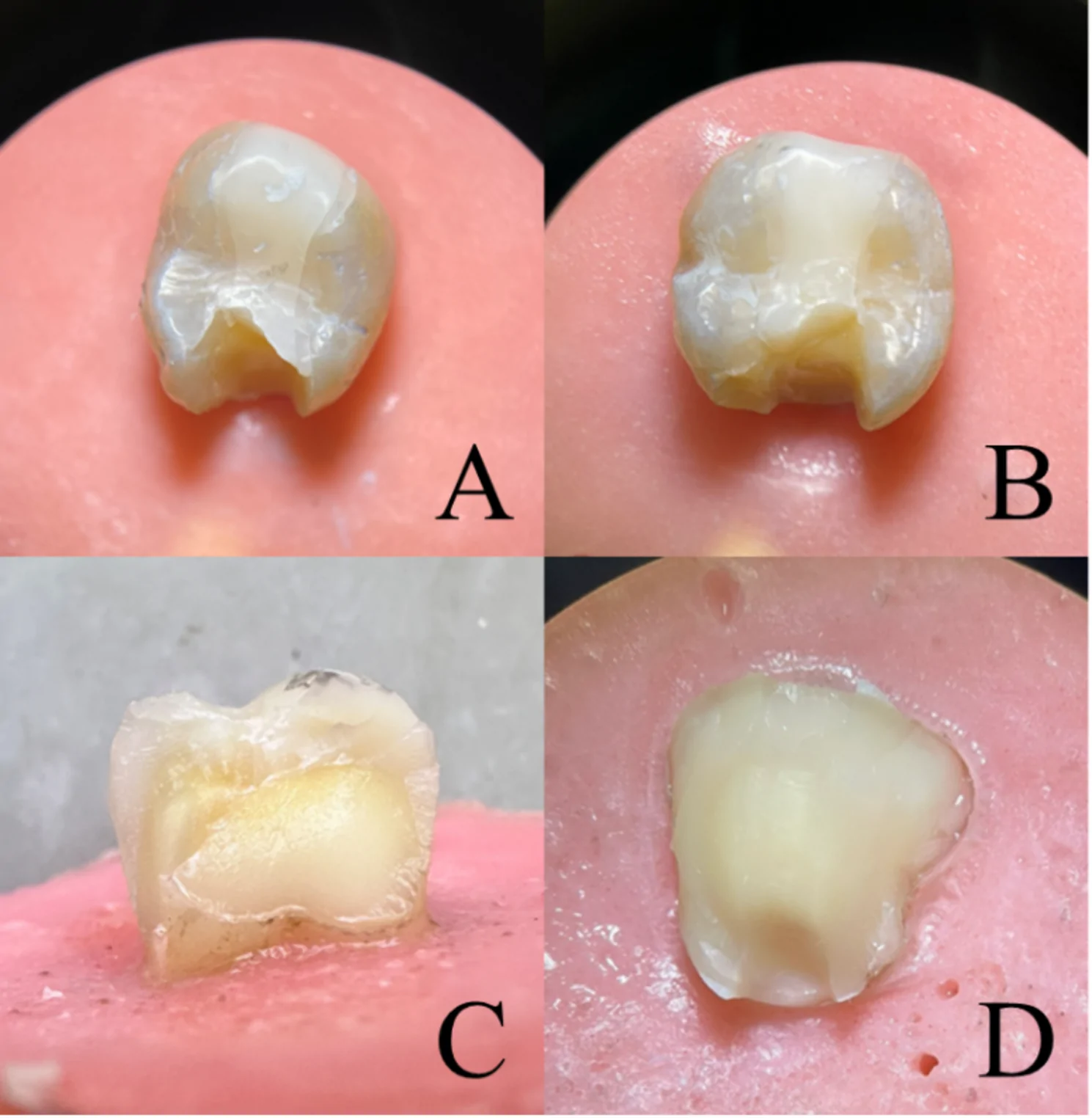
Light microscope images of fracture modes: (**A**) Mode I fracture, (**B**) Mode II fracture, (**C**) Mode III fracture, and (**D**) Mode IV fracture

Fracture modes according to Burke’s classification [22] are shown in Table 5; Fig. 6. Examples of fracture modes are shown in Fig. 7. A higher proportion of catastrophic Mode IV fractures was observed in Group 1, whereas Group 3 demonstrated a greater percentage of favorable Mode I fractures. However, statistical comparison of fracture mode distribution revealed no significant differences among the groups (*p* = 0.749).

**Table 5:** Percentage distribution of fracture modes for each tested group

Group (*n* = 10)	Fracture modes			
	Mode I Fracture (%) (Repairable)	Mode II Fracture (%) (Repairable)	Mode III Fracture (%) (Repairable)	Mode IV Fracture (%) (Irreparable)
Group 1	3 (30%)	3 (30%)	-	4 (40%)
Group 2	4 (40%)	2 (20%)	1 (10%)	3 (30%)
Group 3	6 (60%)	1 (10%)	-	3 (30%)
Total	13 (40%)	6 (20%)	1 (7.5%)	10 (32.5%)

* No statistically significant difference was observed among groups (*p* = 0.749; Fisher–Freeman–Halton test)

## Discussion

The adaptation of resin-based cements is strongly influenced by their viscosity. Low-viscosity cements demonstrate superior penetration into microspaces and form thinner cement layers [23–26]. Conversely, increased viscosity and the presence of residual cement are associated with larger marginal gaps and diminished adaptation over time, primarily due to hydraulic pressure [27, 28]. Resin cements may also serve as shock absorbers during fracture testing [29], whereas adhesive agents enhance dentin sealing, thereby improving both adaptation and fracture resistance [22, 30–32]. Furthermore, preheating resin-based materials has been shown to increase their degree of conversion [33], improve marginal adaptation by reducing viscosity [25, 34, 35], and decrease polymerization shrinkage [36]. This study aimed to compare marginal gap formation at enamel and dentin margins and the fracture strength of inlay restorations luted with preheated resin composite versus conventional dual-cure resin cement. Based on the results of the marginal gap analysis, the first null hypothesis stating that no significant difference exists in marginal gap width between inlays luted with preheated resin composites and those luted with conventional resin cement was rejected. In contrast, the second null hypothesis stating that there is no significant difference in fracture strength between inlays luted with preheated resin composite and those luted with conventional resin cement was not rejected.

Micro-CT has become an increasingly valuable tool in dental research, offering significant advantages over conventional two-dimensional techniques such as scanning electron microscopy (SEM) or dye penetration. Unlike these methods, micro-CT provides a three-dimensional, non-destructive evaluation of the tooth-restoration interface, enabling repeated scanning of the same specimen over time, while preserving the specimen for additional tests. In gap analysis, micro-CT allows the precise measurement of gap volume and distribution across the entire adhesive interface, rather than being limited to cross-sectional observations. It also produces high-resolution images that can reveal topographical differences between enamel and dentin margins. Moreover, micro-CT avoids artifacts introduced by sectioning or specimen preparation, which are inherent in destructive methods [37–39]. Consequently, in this study, micro-CT was selected as the primary method for gap evaluation, providing comprehensive 3D mapping of marginal discrepancies in both direct and indirect restorations. This methodological choice strengthens the reliability of the findings and aligns with recent trends in restorative research that emphasize digital, high-resolution, and reproducible evaluation techniques.

In order to simulate intraoral aging, all restored teeth were subjected to 10,000 thermal cycles (5–55 °C) [40] and 50,000 simulated chewing cycles [41] (100 N loading) to represent one year of clinical functions. Thermal cycling exposes the adhesive interfaces to repeated contraction and expansion stresses, whereas mechanical loading simulates masticatory forces. Together, these aging methods provide valuable insight into the long-term performance of luting materials and restorations under conditions that closely resemble the oral environment.

Heating resin composites to 54–65 °C does not critically raise intrapulpal temperatures [42–44]. However, it can increase monomer mobility, conversion, and crosslinking, while also reducing viscosity and improving handling during luting procedures [45–53]. Nevertheless, previous studies have reported inconsistent results regarding the use of preheated resin composites for luting indirect restorations. Alvarado and others recommended the use of preheated resin composites as luting agents, as they demonstrated better marginal adaptation than resin cements [54]. However, Goulart and others reported that the use of preheated resin composites for luting indirect restorations did not improve adhesion [55]. These inconsistent findings may be partly explained by differences in the rheological behavior of the restorative materials. Although preheating generally reduces composite viscosity and improves adaptation, its effect is highly material-dependent. Thermoviscous composites are specifically engineered to undergo a pronounced reduction in viscosity upon heating, whereas conventional bulk-fill composites exhibit a more limited rheological response [56, 57]. VisCalor Bulk was specifically developed as a thermoviscous composite intended to improve flow after heating. In contrast, Filtek One Bulk Fill was primarily designed as a conventional bulk-fill restorative material with a Bis-GMA-free, AFM-based formulation optimized for stress relief during polymerization rather than thermal flow [44, 58]. Previous studies have shown that thermoviscous composites exhibit greater viscosity reduction after preheating, while separate investigations have reported improved internal adaptation and lower polymerization shrinkage stress for these materials compared with conventional bulk-fill composites [56–59]. In addition, the two restorative composites were preheated using different manufacturer-recommended heating programs, which differed in both target temperature and heating duration. Previous studies have shown that differences in preheating temperature and duration may influence the handling and rheological behavior of resin composites, and that these effects are material-dependent [13, 60]. Therefore, the differences observed between the two preheated composite groups should be interpreted as the combined influence of material composition and their respective heating protocols rather than either factor alone.

In the present study, restorations luted with preheated Filtek One Bulk-Fill exhibited significantly higher gap values at enamel margins compared with conventional resin cement, whereas VisCalor Bulk showed gap values comparable to those using conventional resin cement. These results suggest that preheated resin composites do not behave uniformly and that some materials, such as VisCalor Bulk, may perform similarly to conventional resin cements when used as luting agents. Although both VisCalor Bulk and Filtek One Bulk Fill are categorized as bulk fill resin composites, they differ in filler content and resin matrix composition. These differences may lead to distinct viscosity changes and marginal adaptation during preheating. In general, resin composites contain a higher filler concentration and lower organic matrix than resin cements, which can affect their flow and interfacial integrity [61, 62]. Similarly, previous research has reported that heating Filtek One Bulk Fill to 55 °C resulted in a decreased degree of conversion compared with use at room temperature, likely due to its resin matrix composition and material-specific response to preheating [44]. In addition, differences in the polymerization mechanisms of the luting materials may also have influenced the present findings. Although the resin nano-ceramic CAD/CAM material used in this study allows favorable light transmission, the dual-cure resin cement also benefits from an additional chemical polymerization mechanism that is absent in the preheated restorative composites. Therefore, the higher enamel marginal gap observed for Filtek One Bulk Fill may have been influenced not only by its material composition and rheological behavior after preheating but also by differences in curing mechanisms.

Enamel and dentin represent different bonding substrates, which necessitates their separate evaluation. Enamel, with its high mineral content and homogeneous structure, provides predictable micromechanical retention after acid etching, whereas dentin, due to its higher water and organic content, tubular structure, and outward fluid flow, offers a less reliable bonding surface [63]. Previous studies have shown that enamel margins provide stronger adhesion, whereas dentin margins are more prone to gap formation and microleakage [63–68]. 

The adhesive strategy may also influence bonding performance. In the present study, the universal adhesive was applied using the etch-and-rinse strategy. Although phosphoric acid etching of dentin may increase technique sensitivity, recent evidence indicates that the etch-and-rinse strategy with universal adhesives provides favorable clinical performance [69]. In addition, an experimental study reported comparable dentin bond strength for Futurabond U when applied using either the etch-and-rinse or self-etch strategy, while a systematic review concluded that etch-and-rinse may provide favorable bond strength outcomes with universal adhesives under certain experimental conditions [70, 71]. 

According to the results of the present study, the gap formation values of inlays luted with conventional resin cement were significantly higher at the dentin margins located apically to the CEJ than at the coronally located enamel margins. In cavities where margins are placed apically to the CEJ, the adhesive bond between resin and dentin is more disadvantageous in terms of gap formation. This is because dentin differs morphologically, histologically, and structurally from enamel. Dentin contains a higher proportion of water and organic material [72], making it highly hydrophilic and difficult to infiltrate with hydrophobic adhesives that cannot achieve complete dehydration [73]. In this study, however, inlays luted with preheated resin composites exhibit no observable differences in gap formation between enamel and dentin margins. This may be due to the reduced viscosity of resin composites upon preheating, which improves their adaptation to cavity walls. Thermal effects may facilitate resin monomers sliding over one another, resulting in more effective wetting of cavity walls. Consequently, the cement may overcome the disadvantages associated with dentin’s hydrophilic nature [74]. 

Fracture strength testing was included because it is one of the most widely accepted methods for evaluating the mechanical performance of indirect restorations and the ability of the restored tooth-restoration complex to withstand occlusal loading [18, 19]. In this study, Class II MOD cavities were preferred because the loss of marginal ridges and microcracks caused by occlusal stresses significantly reduce fracture strength; as a result, MOD preparations are considered among the weakest cavity designs in terms of tooth strength [75–77]. The type of luting agent is not considered a primary determinant of fracture strength. In the present study, the use of preheated resin composites—despite their improved mechanical properties—did not result in a significant enhancement in fracture strength compared to conventional resin cement. The intact control group (Group 4) demonstrated significantly higher values than restored groups. Similar findings have been reported in studies evaluating indirect CAD/CAM inlay restorations, in which fracture resistance was primarily influenced by cavity design, remaining tooth structure, and restorative material rather than by the luting procedure alone [78–80]. The limited and conflicting evidence available in the literature also supports cautious interpretation of the present findings. Previous in vitro studies evaluating preheated restorative composites as luting agents for indirect restorations have reported inconsistent fracture strength outcomes, with no clear superiority of either preheated composites or conventional resin cements [11, 20, 81]. Fracture mode analysis demonstrated differences among the restored groups. In the conventional resin cement group, catastrophic Mode IV fractures were most frequent (40%), and Mode II fractures were also commonly observed (30%). These results suggest relatively strong adhesion, but with a tendency toward more unfavorable failure modes. In contrast, the preheated bulk-fill resin composite group showed the highest proportion of favorable Mode I fractures (60%), which may be associated with the higher gap values observed in this group, indicating weaker adhesion at the tooth-restoration interface.

### Limitations

This in vitro study cannot fully replicate intraoral conditions such as long-term aging, saliva, and functional occlusal forces. Moreover, only two preheated resin composites and one resin cement were tested using a single cavity design, which may limit the generalizability of the findings.

Micro-CT analysis was performed on a limited number of specimens due to the high cost and time-intensive nature of the method. Although significant differences were detected, the relatively small sample size may have reduced the statistical power to identify more subtle differences in marginal adaptation and limits the generalizability of the micro-CT findings. Because marginal gap formation constituted one of the primary outcomes of the present study, confirmation of these findings in larger sample sizes would further strengthen the evidence.

The fracture strength test design may have limited sensitivity in detecting differences attributable solely to the luting material, as the tooth structure itself represents the primary load-bearing component. Accordingly, fracture mode findings should be interpreted with caution, as no statistically significant differences were observed among the groups (*p* = 0.749).

One limitation associated with preheated resin composites is the rapid decrease in temperature during clinical manipulation, which may reduce the viscosity-related benefits achieved immediately after heating. To minimize this effect, a VisCalor dispenser was used in the present study to provide rapid heating immediately before placement and to maintain a clinically relevant working time.

In addition, the selection of different composite materials and heating protocols was influenced by the technical compatibility requirements of the heating device, which is designed to accommodate compule-based systems. As a result, materials from different manufacturers were used according to their respective manufacturer-recommended heating protocols. This may have introduced confounding variables that limit the direct attribution of the observed effects solely to the preheating process, since heating temperature and duration may influence the rheological behavior and handling characteristics of resin composites in a material-dependent manner. Therefore, the differences observed between the two preheated composite groups should be interpreted as reflecting the combined influence of material composition and manufacturer-recommended heating protocols rather than material composition alone.

To minimize variability, standardized MOD cavity preparations, controlled thermomechanical aging, and consistent luting protocols were applied across all groups using sound teeth with similar morphology.

Future studies should investigate a broader range of restorative materials and cavity designs under extended thermomechanical aging protocols. In addition, in vivo clinical trials are required to confirm the clinical applicability of preheated resin composites for luting indirect restorations.

## Conclusion

Within the limitations of this in vitro study, preheated resin composites demonstrated marginal adaptation and fracture strength comparable to those of conventional resin cement, indicating their potential use as alternative luting materials for indirect restorations. However, differences in marginal gap formation were observed among the tested preheated composites, suggesting that the performance of preheated composites is material-dependent and not all formulations provide equivalent marginal outcomes.

## Data Availability

The datasets generated and/or analysed during the current study are available from the corresponding author on reasonable request.
